# Optimal strategy of primary percutaneous coronary intervention for acute myocardial infarction due to unprotected left main coronary artery occlusion (OPTIMAL): study protocol for a randomised controlled trial

**DOI:** 10.1186/s13063-019-3211-0

**Published:** 2019-03-08

**Authors:** Yang Gao, Feng Zhang, Chenguang Li, Yuxiang Dai, Ji’e Yang, Ya’nan Qu, Juying Qian, Junbo Ge, for the OPTIMAL trial investigators

**Affiliations:** Department of Cardiology, Zhongshan Hospital, Fudan University; Shanghai Institute of Cardiovascular Diseases, 180 Fenglin Road, Shanghai, 200032 China

**Keywords:** Deferred stent implantation, Percutaneous coronary intervention, Acute myocardial infarction

## Abstract

**Background:**

Primary percutaneous coronary intervention (PCI) for patients presenting with acute myocardial infarction (AMI) caused by left main coronary artery occlusion is associated with significantly higher mortality and risks of major adverse cardiovascular events. Deferred stent implantation may improve prognosis of primary PCI by reducing distal embolization and no-reflow phenomenon. There is no randomized clinical trial focusing on the effect and outcome of deferred stent implantation on primary PCI for left main coronary artery occlusion in contrast with conventional strategy.

**Methods:**

The Optimal Strategy of Primary Percutaneous Coronary Intervention for Acute Myocardial Infarction due to Unprotected Left Main Coronary Artery Occlusion (OPTIMAL) study (ClinicalTrials.gov Identifier: NCT03282773) is an open-label, randomized, multicenter clinical trial in which 480 patients presenting with AMI caused by left main coronary artery occlusion recruited from 30 hospitals in mainland China will be randomly assigned 1:1 to immediate stenting or deferred stenting (scheduled 4–10 days after primary angioplasty) groups. The primary endpoint is a composite of all-cause mortality or recurrent myocardial infarction at 30 days after randomization. The secondary outcomes include all-cause mortality, cardiac death, recurrent myocardial infarction, and unplanned target vessel revascularization at 30 days, 6 months, and 12 months.

**Discussion:**

The OPTIMAL study is designed to compare the clinical performance of deferred stenting with that of immediate stenting for AMI caused by left main coronary artery occlusion.

**Trial registration:**

ClinicalTrials.gov Identifier: NCT03282773. Registered on 10 September 2017.

**Electronic supplementary material:**

The online version of this article (10.1186/s13063-019-3211-0) contains supplementary material, which is available to authorized users.

## Background

Of all patients with acute myocardial infarction (AMI), 4–7% have a culprit lesion in the left main coronary artery whose abrupt occlusion triggers ischemia and myocardial infarction (MI) [1, 2]. Unprotected left main coronary artery occlusion–induced AMI (LM-AMI) often presents with cardiogenic shock and is associated with higher risks of major cardiac adverse events and higher mortality even if treated with reperfusion therapy in time [3, 4]. Coronary artery bypass graft (CABG) is recognized as the standard revascularization strategy for this situation [5, 6]. Nonetheless, although CABG is recommended by the guidelines, it still carries very high mortality in patients with LM-AMI. Percutaneous coronary intervention (PCI) for LM-AMI was revealed to have a clinical outcome comparable to that of CABG in recent studies because of the application of second-generation drug-eluting stents and improvement of PCI procedure [7]. Primary PCI (PPCI) for LM-AMI is an alternative revascularization strategy to CABG for selected patients.

Stents are immediately implanted in conventional PPCI after successful revascularization, and 12–30% of patients underwent immediate stenting have not achieved thrombolysis in myocardial infarction (TIMI) grade 3 flow, which usually indicates impaired perfusion and unfavorable clinical outcome [8]. Impaired perfusion or no-reflow phenomenon, partly explained by distal embolization and microvascular obstruction caused by stent implantation, may induce increased infarct area, reduced ventricular function, and poor prognosis [9]. In addition, no-reflow–triggered ischemia reperfusion injury after immediate stenting may cause ventricular arrhythmia, myocardial stunning, and even sudden death [10, 11]. Deferred stent implantation, as an alternative strategy, can attenuate distal embolization and reperfusion injury since enhanced anti-thrombotic therapies and myocardial preconditioning of oxidative stress can be applied during the delayed period. On the other hand, deferred stent implantation provides doctors with time to apply anti-thrombotic therapy to alleviate thrombus burden and optimize stent implantation. The efficacy of deferred stenting was supported by some studies with improved peri-procedure outcome and potential favorable long-term prognosis [12–16]. In contrast, several studies, including three randomized controlled trials (RCTs), failed to prove the advantage of delayed stenting and even led to controversial conclusions [17–19]. Further investigation is necessary to evaluate the safety and efficacy of deferred stent implantation for LM-AMI. Therefore, the present study is aimed to compare deferred stent implantation with conventional procedure and optimize the standard PCI procedure for patients with LM-AMI.

## Methods

### Objectives and study design

The aim of this open-label, multicenter RCT is to compare immediate stenting with deferred stenting for LM-AMI in 30 hospitals in China. We hypothesize that patients with LM-AMI receiving deferred stent implantation will have improved short-term and long-term clinical outcomes compared with those receiving immediate stenting. A total of 480 patients with diagnosed LM-AMI will be randomly assigned in a 1:1 ratio to either deferred stenting or immediate stenting and followed for up to 12 months.

### Recruitment

Patients with LM-AMI will be consecutively enrolled from 30 clinical centers in China. We recruited participating centers on a voluntary basis. Four centers were not included in the trial, because the numbers of PPCI per year in these centers were less than 50 and they may not have enough experience in treating LM-AMI. The participating centers are located mostly in southern China (20 out of 30) while several other hospitals (10 out of 30) from northern districts are also involved (Additional file 1: Table S1). Most of them are third-grade class-A hospitals (25 out of 30). All participating centers can perform PPCI in 24 h, 7 days a week, and had a minimum volume of 500 PCI procedures and 50 PPCI procedures annually. Coronary care unit and surgery backup are available in all of the centers involved. Additionally, chest pain centers and quick-reaction systems of PCI for patients with AMI have been established among all of these centers. The study protocol and documents have been distributed to the investigators of each center. The OPTIMAL (Optimal Strategy of Primary Percutaneous Coronary Intervention for Acute Myocardial Infarction due to Unprotected Left Main Coronary Artery Occlusion) staff of physicians completing the protocol are responsible for online training and problem solving. Recruitment started on November 1, 2017 and will terminate when the estimated number of patients is reached and we estimate that this will take 3-4 years. Patients are eligible for enrollment if AMI is diagnosed within 12 h and primary angiography shows left main coronary artery occlusion and they reach TIMI 3 flow after reperfusion treatment of balloon dilatation or optional thrombus aspiration or both (Table 1) [20]. Patients with AMI including ST-segment elevation myocardial infarction (STEMI) and non-ST-segment elevation myocardial infarction (NSTEMI) will be enrolled in the study because a certain number of patients with left main coronary artery occlusion present with NSTEMI instead of STEMI. Patients who are more than 80 years old and have malignant tumor, end-stage organ failure, or other terminal diseases are excluded from the study for their shorter life expectancy, more clinical complications, and less availability to complete the follow-up. In addition, patients who cannot tolerate PPCI and anti-platelet therapy or who are included in other ongoing trials will not be eligible. Both peri-procedural and long-term outcomes will be recorded in the 12-month follow-up. Written informed consent is obtained before primary angiography.

**Table 1:** Eligible criteria

Inclusion criteria	
• 18 years old ≤ age ≤ 80 years old	
• Clinical diagnosis of acute myocardial infarction (AMI) occurred within 12 h^a^	
• Left main coronary artery occlusion—thrombolysis in myocardial infarction (TIMI flow 0, 1, or 2)—confirmed by primary angiography	
• TIMI flow grade 3 achieved after interventional pretreatment such as thrombus aspiration and balloon dilatation	
Exclusion criteria	
• Life expectancy less than 1 year^b^	
• Contraindications to aspirin or other anti-platelet drugs	
• Patients who are included in other ongoing trials	
• Pregnant	
• Patients unable or unwilling to sign the informed consent form	

^a^AMI is defined in accordance with the *Third Universal Definition of Myocardial Infarction* [20]

^b^Life expectancy here is the mean number of years of life remaining, which is estimated through the medical history and clinical parameters by the investigators of each center. This criterion is suitable only for patients who have a malignant tumor, end-stage organ failure or other terminal diseases. Patients with medical history of these terminal diseases and life expectancies assessed by specialists of less than 1 year are not eligible

### Randomization

**Fig. 1:**
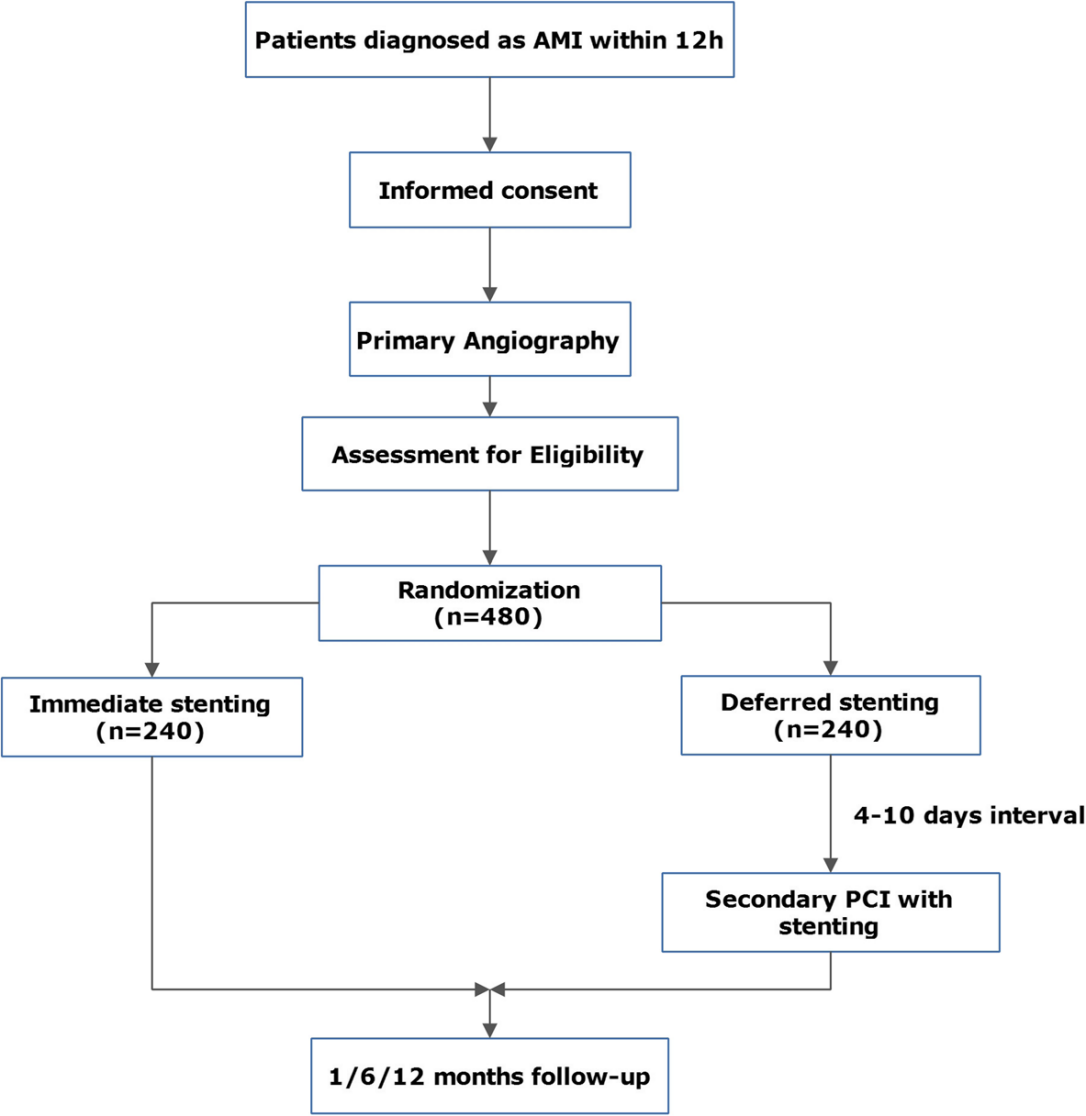
OPTIMAL study design flowchart. Abbreviations: *AMI* acute myocardial infarction, *OPTIMAL* Optimal Strategy of Primary Percutaneous Coronary Intervention for Acute Myocardial Infarction due to Unprotected Left Main Coronary Artery Occlusion

Eligible patients will be randomly assigned 1:1 to immediate stenting or deferred stenting after primary angioplasty and informed consent (Fig. 1). Random allocation sequence has been generated by using a computer-based system before recruitment started, and results are encrypted and uploaded to a network accessible to all research centers routinely in stacks of sealed electronic envelopes that can be uncovered only with passwords when patients are enrolled. The block size is 4. When a patient is eligible in one center, the investigators of that center will download and open the electronic envelope with passwords obtained from the system and get the result of random allocation and will report the information of enrollment in an online research database which will inform all of the other investigators.

### Clinical procedures

PCI procedures are performed in accordance with each clinical center’s standard routines. There are no restrictions on the balloons, guide wires, thrombus aspiration, or intra-aortic balloon pump usage. The choice of transradial or transfemoral approach will be determined by the operator. We recommend that the participating centers apply the minimalist immediate mechanical intervention (MIMI) technique with small balloon dilatation and thrombus aspiration for the pretreatment. New-generation drug-eluting stents are applied in all participating centers, and the exact type and size of the stents are possibly different. For immediate stenting, stents are implanted immediately after blood flow is regained by interventional pretreatment. Deferred stenting is scheduled 4–15 days after primary angioplasty and in the same hospitalization period. Immediate stenting will be applied if TIMI grade 3 flow cannot be retrieved and these cases will not be enrolled in analysis. CABG is considered as an alternative and will be scheduled within 6 h after PCI procedure failure. For the deferred stenting group, stent implantation will be canceled at the operator’s discretion if the patient is unsuitable for deferred stenting or stent implantation is unnecessary during the second PCI. These patients will be excluded from the per-protocol set but will still be in the intention-to-treat set.

All patients will be transferred to the coronary care unit after the primary angiographic procedure. Intravenous glycoprotein IIb/IIIa inhibitor will be maintained for 18–36 h after PPCI. A loading dose of aspirin and P2Y12 inhibitors will be given before the procedure. An intravenous bolus of unfractionated heparin (100 U/kg) will be administrated right before the procedure to achieve therapeutic activated clotting time. Dual-anti-platelet therapy will be maintained during the deferred period and for at least 1 year after PCI. The peri-procedural treatment is in accordance with the Chinese guidelines for the management of AMI [21].

### Sample size

The annual primary outcome event rate is 22.9%, which we estimated through integrating results of several observational trials [7, 22–26]. Because research data about stent implantation in patients with LM-AMI cannot be found, we have to use the data from other trials that enrolled only a few patients with LM-AMI. According to the results of the OPTIMAL study and the experience of our center, we determined a 35% relative decrease in primary outcome, which is conservative given the high mortality and incidence of adverse events for LM-AMI [15]. The study planned to enroll 240 patients for each arm given a 10% rate of loss (two-sided alpha of 0.05 and 80% power to demonstrate the relative decrease).

### Follow-up

**Fig. 2:**
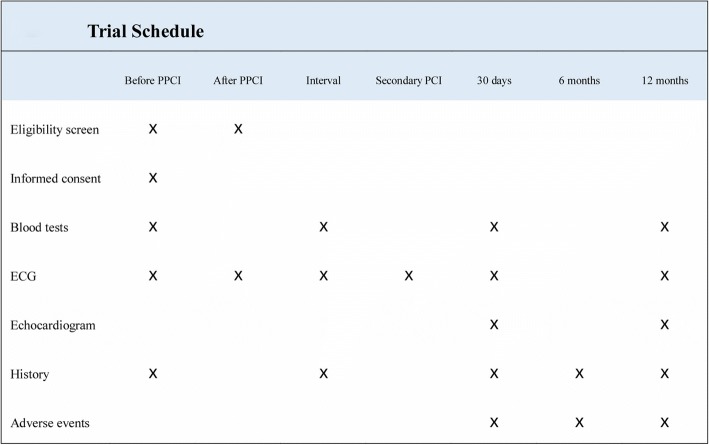
Time schedule of the study. Abbreviation: *PPCI* primary percutaneous coronary intervention

Both peri-procedural and mid-term clinical outcomes are included in the follow-up plan (Fig. 2). All patients will be followed for 12 months. Patients will receive a mail message and phone call 2 weeks in advance to remind them of the time point for following up. The SPIRIT (Standard Protocol Items: Recommendations for Interventional Trials) checklist is in presented in Additional file 2.

### Outcomes

#### Primary endpoints

Since the study is focused on the optimal interventional strategy for patients with LM-AMI in order to reduce the mortality and incidence of adverse events, the primary endpoint is a composite of all-cause mortality or recurrent MI at 30 days after PPCI.

#### Secondary endpoints

Secondary endpoints include all-cause mortality, cardiac death, recurrent MI, and unplanned target vessel revascularization. The time frame and methods of data acquirement are shown in Table 2. All clinical events relating to endpoints are reported to and adjudicated by an independent clinical endpoints committee, which consists of five cardiologists not participating in this trial. The definitions of endpoints are clarified in Additional file 3: Table S2.

**Table 2:** Time frame and data collection of primary and secondary outcomes

Endpoints	Composition	Time frame	Data collection
Primary endpoints	All-cause mortality or recurrent myocardial infarction (MI)	30 d	History
Secondary endpoints	All-cause mortality	30 d/6 m/1 y	History
	Cardiac death	30 d/6 m/1 y	History
	Recurrent MI	30 d/6 m/1 y	History
	Unplanned target vessel revascularization	30 d/6 m/1 y	History

### Data collection and management

Patients’ baseline characteristics are collected with case report forms (CRFs) during hospitalization at least 24 h after PPCI by investigators of each center. Angiographic data are recorded in the PCI procedure. According to the follow-up plan, patients are required to undergo recording of history and echocardiogram in each center at 1- and 12-month follow-up.

Data as outlined above will be collected with electronic CRFs by investigators and research coordinators and transferred to an encrypted online database operated by Zhongshan Hospital. The original CRFs and other records of data will be uploaded to the database. Data usage adheres to local laws and regulations of participating centers. Patient privacy is protected by restricting the access to dataset to relevant individuals—investigators, statistical analyzers, clinical research associates (CRAs), and representatives of the ethics committee—and replacement of the actual names in the documents with serial numbers. Representatives of participating centers are responsible for collecting data of enrolled patients in that center and uploading to the database. Angiographic and echocardiographic data are stored in CD-ROMs and sent to a core lab in Zhongshan Hospital, where the data are analyzed by specialists blinded to random allocation.

### Statistical analyses

Numerical data are summarized with means and standard deviations or medians with interquartile ranges depending on the distribution. Categorical data were described as proportions and percentages. Comparisons of numerical variables are conducted with the Student *t* test. Categorical variables and outcomes were compared by using the chi-squared test or Fisher exact test. All tests were two-tailed with a significant level of 0.05. Logistic regression is applied to adjust the significant variables in baseline data between the two groups and assess the outcomes presented with odds ratios and 95% confidence intervals. All data are analyzed by independent statisticians blinded to the random allocation using Stata version 14.0 and SAS version 9.4 (or higher versions of these programs).

The statistical analyses of primary and secondary outcomes will be conducted in both the intention-to-treat set and the per-protocol set. According to the intention-to-treat principle, all of these eligible patients who actually did not receive deferred stenting will still be included in the intention-to-treat analysis. In the per-protocol set, patients who undergo randomization but withdraw consent to participate or not treated according to the allocated procedure after randomization will be excluded from per-protocol statistical analyses.

### Study management

An independent CRA will monitor the study process and verify protocol compliance every 6 months. The CRA has access to all documents in the database, including original medical history, images, and CRFs. Data correctness will be assessed and serious adverse events will be reported to the ethics committee of the particular center and Zhongshan Hospital. The ethics committee of Zhongshan Hospital has the authority to terminate the trial according to local laws or institutional regulations. The investigators should keep the participating centers, ethics committee, and the journal informed of any major protocol modifications.

### Ethical considerations

This study is conducted in accordance with the ethical principles of the Declaration of Helsinki (2013). The protocol of this study has been read and approved by the ethics committee of participating hospitals. Printed informed consent and detailed information about the study will be offered to patients before randomization. The safety and feasibility of deferred stent implantation have been confirmed by several trials in patients with AMI [14, 15, 18]. All information and data of this trial are encrypted and stored in an online database accessible only to main researchers and administrators.

Patients primarily enrolled have rights to withdraw at any time point and the reasons will be documented. If patients in the deferred stenting group decided to withdraw between primary angiography and secondary PCI, stent implantation or CABG is still available for them while follow-up will be cancelled. In addition, PCI or CABG is ready for any patients presenting with recurrent ischemia and MI after stent implantation in the hospital.

## Discussion

The main objective of the OPTIMAL study is to compare the clinical outcome of deferred stent implantation with that of conventional immediate stenting. Deferred stenting is recommended by some investigators as an alternative strategy considering relatively reduced distal embolization, intraprocedural thrombotic complications, and no-reflow [14].

Distal embolization after PCI, which is visible on the coronary angiogram in 15.2% of patients, is attributed to crushing of lesion or thrombus in the culprit lesion [9, 27, 28]. Patients with distal embolization are more likely to have reduced myocardial blush grade and TIMI reperfusion grade after angioplasty, suggesting worse reperfusion [29, 30]. Some previous studies found increased mortality rates at 1- and 5-year follow-up of patients with distal embolization and an increased risk of heart failure regardless of the use of distal protection [9, 31–33].

A high thrombus burden is highly predictive of microvascular embolization, no-reflow phenomenon, and consequently greater infarct size and worse prognosis after PPCI [9, 34, 35]. The thrombus burden can be ameliorated to some extent through anti-thrombotic and anti-coagulant therapy, which will ease stent deployment and perfusion restoration. In particular, left main coronary occlusion is usually accompanied by heavy thrombus burden and slow-flow or no-reflow caused by embolization of distal vessels involving a large scale of myocardium as well as severe ischemia reperfusion injury. PCI for left main coronary artery occlusion requires larger stents and balloons, which sometimes cause more crushing and emboli. Embolism of left anterior descending or circumflex will lead to MI and additional angioplasty. For patients with cardiogenic shock and undergoing PPCI, which is frequent (26%) [36] when the left main coronary artery is occluded, deferred stent implantation can improve the perfusion and cut down time and contrast-agent consumption. Deferred stent implantation, as a strategy to reduce complications, is probably better than immediate stenting in LM-PCI.

There have been several randomized or non-randomized clinical trials regarding deferred stenting for patients with AMI. Meta-analyses of these studies confirmed better procedure-related angiographic events and lower risk of peri-procedural composite events and abnormal flow in patients undergoing PPCI for AMI [37, 38]. However, no significant improvement of long-term outcome has been revealed. In contrast, the deferred stenting group presents with an increased rate of target vessel revascularization and greater microvascular obstruction size in DANAMI 3-DEFER and MIMI studies [17, 19]. There are few LM-AMI patients in these trials, and we assume that patients with LM-AMI would get more benefit from deferred stent implantation because PPCI for LM-AMI is often associated with more severe distal embolization, reperfusion injury, or cardiogenic shock, together with increased mortality. Furthermore, it has been pointed out that a deferred stent implantation for a mean of no more than 48 h, as in the DANAMI 3-DEFER and MIMI studies, is probably not enough for spontaneous structural modifications and anti-thrombotic treatment to take effect in all cases [39]. In this study, a minimal delay of 4 days is taken to ensure enough time for the therapy, and we believe that intense anti-thrombotic treatment can alleviate thrombus burden and enable better stent apposition. The deferred interval is decided considering the effect of deferred stenting and practical limitations (for example, the hospital stay). We are also interested in the long-term left ventricular function in echocardiogram and myocardial salvage detected by magnetic resonance imaging, as have been revealed in previous studies on non-LM-AMI patients [14, 16, 17], even without a reduction in microvascular obstruction in the DANAMI 3-DEFER study.

## Trial status

Recruitment started in November 2017 and is estimated to be completed in December 2020.

## Additional files

Additional file 1: **Table S1.** A list of the participating hospitals. (DOCX 24 kb)

Additional file 2: SPIRIT (Standard Protocol Items: Recommendations for Interventional Trials) 2013 Checklist: Recommended items to address in a clinical trial protocol and related documents*. (DOC 124 kb)

Additional file 3: **Table S2.** Definitions of endpoints. (DOCX 18 kb)

## Abbreviations

AMI: Acute myocardial infarction
CABG: Coronary artery bypass graft
CRA: Clinical research associate
CRF: Case report form
LM-AMI: Left main acute myocardial infarction
MI: Myocardial infarction
MIMI: Minimalist immediate mechanical intervention
NSTEMI: Non ST-segment elevation myocardial infarction
OPTIMAL: Optimal Strategy of Primary Percutaneous Coronary Intervention for Acute Myocardial Infarction due to Unprotected Left Main Coronary Artery Occlusion
PCI: Percutaneous coronary intervention
PPCI: Primary percutaneous coronary intervention
RCT: Randomized controlled trial
STEMI: ST-segment elevation myocardial infarction
TIMI: Thrombolysis in myocardial infarction
