# Supplementary material for: Optimal strategy of primary percutaneous coronary intervention for acute myocardial infarction due to unprotected left main coronary artery occlusion (OPTIMAL): study protocol for a randomised controlled trial
Source: Trials. 2019 Mar 8;20:162. doi: 10.1186/s13063-019-3211-0 (PMC6408768; doi:10.1186/s13063-019-3211-0)
Supplement: Supplementary file 1 — Table S1. A list of the participating hospitals. (DOCX 24 kb) [file 13063_2019_3211_MOESM1_ESM.docx]

Table S1. A list of the participating hospitals.

| Name | City | Location^1^ | Class^2^ | Bed size | Annual  AMI patients^3^ |
| --- | --- | --- | --- | --- | --- |
| The People’s Hospital of LiaoNing Province | Shenyang | North | 3A | 280 | 422 |
| The General Hospital of Shenyang Military | Shenyang | North | 3A | 160 | 305 |
| Xuzhou Central Hospital | Xuzhou | North | 3A | 326 | 651 |
| Xinxiang Central Hospital | Xinxiang | North | 3A | 190 | 260 |
| Bethune International Peace Hospital | Shijiazhuang | North | 3A | 110 | 280 |
| The Second People’s Hospital of Lianyungang | Lianyungang | North | 3A | 80 | 136 |
| Liaocheng People’s Hospital | Liaocheng | North | 3A | 180 | 230 |
| Liaoyang Central Hospital | Liaoyang | North | 3A | 95 | 113 |
| Second Affiliated Hospital of Xuzhou Medical University | Xuzhou | North | 3A | 160 | 198 |
| Linqing People’s Hospital | Linqing | North | 2A | 86 | 150 |
| ZhongShan Hospital | Shanghai | South | 3A | 330 | 486 |
| Shanghai Chest Hospital | Shanghai | South | 3A | 200 | 105 |
| Changhai Hospital | Shanghai | South | 3A | 110 | 300 |
| Shanghai Tenth People’s Hospital | Shanghai | South | 3A | 182 | 355 |
| Shanghai Seventh People’s Hospital | Shanghai | South | 3A | 90 | 120 |
| The First Affiliated Hospital of Anhui Medical University | Hefei | South | 3A | 93 | 145 |
| The Second Affiliated Hospital of Zhejiang University School of Medicine | Hangzhou | South | 3A | 180 | 360 |
| Anhui Provincal Hospital | Hefei | South | 3A | 290 | 402 |
| Wuxi People’s Hospital | Wuxi | South | 3A | 163 | 310 |
| Wuxi Second People’s Hospital | Wuxi | South | 3A | 110 | 210 |
| Taizhou People’s Hospital | Taizhou | South | 3A | 120 | 192 |
| Huangshan City People’s Hospital | Huangshan | South | 3A | 74 | 175 |
| Sir Run Run Hospital, Nanjing Medical University | Nanjing | South | 3A | 54 | 76 |
| Affiliated Hospital of Zunyi Medical College | Zunyi | South | 3A | 147 | 187 |
| Subei People's Hospital of Jiangsu province | Yangzhou | South | 3A | 123 | 204 |
| Taixing People’s Hospital | Taixing | South | 3B | 68 | 201 |
| Changzhou Wujin People’s Hospital | Changzhou | South | 3B | 78 | 106 |
| The People’s Hospital of Chuxiong Yi Autonomous Prefecture | Chuxiong | South | 3A | 85 | 98 |
| Danyang People’s Hospital | Danyang | South | 3B | 60 | 147 |
| Nantong Rich Hospital | Nantong | South | 3B | 72 | 51 |

1.The boundary between northern and southern region is Qingling-Huaihe Line. All participating centers are located in urban areas.

2.3A=Third-grade class-A. Third grade is the highest grade and class-A is the best class of hospitals in China. 3A is the highest level a hospital can achieve.

3. The number of annual AMI patients is an average of numbers in 2016 and 2017 in each participating center.
