# Supplementary material for: Optimal strategy of primary percutaneous coronary intervention for acute myocardial infarction due to unprotected left main coronary artery occlusion (OPTIMAL): study protocol for a randomised controlled trial
Source: Trials. 2019 Mar 8;20:162. doi: 10.1186/s13063-019-3211-0 (PMC6408768; doi:10.1186/s13063-019-3211-0)
Supplement: Supplementary file 3 — Table S2. Definitions of endpoints. (DOCX 18 kb) [file 13063_2019_3211_MOESM3_ESM.docx]

| Table S2. Definitions of endpoints | | |
| --- | --- | --- |
| Endpoints | | Definition |
| Primary Endpoints | All-cause mortality | Numbers of death/Numbers of patients |
|  | Recurrent MI | Recurrent MI is defined according to *Third Universal Definition of Myocardial Infarction (2012)** |
| Secondary  Endpoints | All-cause mortality | See above |
|  | Cardiac death | Incidence of death caused by CAD, structural heart disease or arrhythmias |
|  | Recurrent MI | See above |
|  | Unplanned target vessel revascularization | Incidence of revascularization (PCI or CABG) in target vessel that is not planned by the trial |

* Recurrent MI in this study includes both recurrent MI and re-infarction described in *Third Universal Definition of Myocardial Infarction (2012)*, as the difference is only the timeframe (recurrent MI>28days, re-infarction≤28days).
